# Comparison of forages’ digestion levels for different in vitro *digestion* techniques in horses

**DOI:** 10.1002/vms3.1373

**Published:** 2024-02-18

**Authors:** Kanber Kara, Abdullah Altınsoy

**Affiliations:** ^1^ Department of Animal Nutrition and Nutritional Diseases Faculty of Veterinary Medicine Erciyes University Kayseri Türkiye; ^2^ Health Sciences Institute Department of Animal Nutrition and Nutritional Diseases Erciyes University Kayseri Türkiye

**Keywords:** digestive tract, horse, in vitro digestion, organic matter, short‐chain fatty acids

## Abstract

**Background:**

Forages are widely used in horse diets. Different in vitro techniques are being tried to determine the fermentation levels of forages in the horse digestive tract.

**Objectives:**

This study aimed to evaluate the digestion levels of four dry forages commonly used in horse nutrition: alfalfa herbage, meadow hay, wheat straw, and Italian ryegrass. In vitro total digestion (TDT), in vitro Sunvold–large intestine digestion (SDT) and in vitro Menke–large intestine digestion (MDT) techniques were compared.

**Methods:**

The study determined in vitro true dry matter digestion (T‐DMD), in vitro true organic matter digestion (T‐OMD) and in vitro true neutral detergent fibre digestion (T‐NDFD). Additionally, concentrations of straight short‐chain fatty acids (SCFAs; acetic acid – AA, propionic acid , butyric acid, and valeric acid ) and branched short‐chain fatty acids (BSCFA) were assessed.

**Results:**

The highest in vitro T‐DMD, T‐OMD and T‐NDFD values were determined by the in vitro TDT for the four forages (*p* < 0.05). In vitro T‐DMD and T‐OMD values of alfalfa herbage were higher than those of Italian ryegrass, meadow hay and wheat straw in the in vitro TDT (*p* < 0.001). In addition, in vitro T‐DMD and T‐OMD values of alfalfa herbage in the in vitro SDT were higher than those of meadow hay and wheat straw (*p* < 0.001). In the in vitro TDT, the molarity of AA, total SCFA and BSCFA in the digestion fluid of alfalfa herbage was higher than those of other forages (*p* < 0.05).

**Conclusion:**

The in vitro total enzymatic + fermentative digestion technique for horse forages revealed higher values than the in vitro fermentative digestion techniques. In general, the higher the non‐structural carbohydrate and crude protein contents in the forage, the higher the results of the in vitro TDT compared to the other techniques.

## Graphical abstract

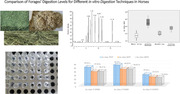

## 1 INTRODUCTION

Horses are monogastric herbivores and forage should be the primary component of their diet. The use of less than the necessary forage negatively affects the gastrointestinal and, subsequently, the general health of the horse (National Research Council [NRC], 2007). The high‐starch horse diet (containing ≥40% of starch‐rich concentrates or ≥starch intake of 2 g/kg BW/meal) (Jansson & Lindberg, 2012; Raspa, Tarantola et al., 2022) changes in the horse gut environment (Raspa, Vervuert, et al., 2022) and causes gastrointestinal disorders such as gastric ulcers and colic (Vondran et al., 2016). The digestion levels of forage in horses vary according to the composition of carbohydrate monomers and bond structures (Cichorska et al., 2014). The digested components of the feed consumed by horses are enzymatically broken down in the stomach and small intestine and absorbed as monomers. However, the fermented components (fibrous feedstuffs, resistance starch and other dietary fibre substances) of the horse ration are degraded by the fermentation of the microorganism's enzymes in the large intestine (especially the cecum and ventral colon), and the fermentation end products are absorbed from there (Hintz & Cymbaluk, 1994). The nutritional value of forage depends on the level of nutrients and end products (straight or branched short‐chain fatty acids: SCFA and BSCFA) in the large intestine of horses and their absorption from enterocytes (Frape, 2004; Julliand & Grimm, 2017). In addition, horses fed with entirely forage or roughage‐based diets can have an improved welfare by increasing the time they spend engaged in feeding behaviour and reducing the frequency of behavioural activities (excitable etc.) (Raspa, Vervuert, et al., 2022).

The forage of horses, herbivores whose rations consist of plant feedstuffs, is fermented in the large intestine and the enzymes produced by the microbial structure change the digestibility of the consumed feed. Especially in the large intestine, the microorganisms responsible for fermentation are *Ruminococcus* spp., *Streptococcus* spp., *Lactobacillus* spp. and *Enterococcus* spp., and their end products (such as gases, SCFA) emerge. The lumen microbial content in the stomach of horses is *Firmicutes* (*Lactobacillus* spp., 108–109 CFU/mL), *Bacteroidetes* (*Prevotella* spp.), and *Proteobacteria* (*Actinobacillus* spp.) (Al Jassim & Andrews, 2009; Dicks et al., 2014; Perkins et al., 2012). It may be inaccurate for forage to be fully understood only by in vitro fermentation in the large intestine of forage digestion in horses or to interpret only based on the in vitro forage digestion of the large intestine (Gandarillas et al., 2021; Kholif et al., 2015; Kujawa et al., 2020). For this reason, the digestion of other nutrients in the stomach and small intestine, as well as the mono and oligosaccharides in the forage content, may change the fermentation degree of the large intestine of beta‐glucan, pectin and gum, hemicellulose and cellulose, which are plant cell wall elements.

The advantages of in vitro methods used to determine the digestion of feeds in horses, such as being low in cost, being fast and reproducible, being carried out in a controlled environment and requiring fewer animals than using the excrement of the animal directly, show its superiority over in vivo methods (Kitessa et al., 1999). However, although the results obtained in in vitro studies largely reflect the studies conducted on live animals, it is necessary to reach a conclusion through repeated live animal experiments in order to ensure the accuracy of the results. At the same time, the procedure of the in vitro method used, and the content and duration of the buffers used may vary, and the results obtained in each in vitro method may differ. In vitro digestion trials are the trials that give reliable results after chemical analyses to determine the feed ingredients. Different in vitro digestion techniques are used to determine the digestion of forage in horses, and there is still no valid method.

This study hypothesizes that digestibility and digestion end products of forage commonly used in horses will give different results in different in vitro methods, and the differences between the methods will change the researchers’ interpretation of forage. In the present study, Hervera et al. (2007)’s omnivore dogs were based on the digestion channel, which in turn was based on the digestion technique based on horses’ ‘in vitro total digestion technique’ (in vitro TDT) enriched with large intestinal fermentation based on the ‘in vitro Sunvold‐large intestine digestion technique’ (in vitro SDT) (Sunvold et al., 1995) and ‘in vitro Menke‐large intestine digestion technique’ (in vitro MDT), adapted from ruminants (Theodorou et al., 1994; Menke et al., 1979). The cost of forage may vary depending on the nutrient content and digestibility, and the preferences of animal owners vary with cost‐effectiveness (Karimi et al., 2022). Another hypothesis of the study is that the digestion capacities of forages used in horse nutrition may give different results in in vitro digestion experiments according to differences in crude protein (CP), neutral detergent fibre (NDF), and non‐structural carbohydrate (NSC) values, major nutritional elements. The aim of this study is to compare the digestion values of alfalfa herbage, meadow hay, wheat straw, and Italian ryegrass, which are widely used as dry forage in horse nutrition, using three different in vitro digestion techniques (in vitro TDT, in vitro MDT and in vitro SDT). In vitro true dry matter digestion (T‐DMD), in vitro true organic matter digestion (T‐OMD) and in vitro true neutral detergent fibre digestion (T‐NDFD) of fibrous feedstuffs and the concentrations of straight SCFA (acetic acid (AA), propionic acid (PA), butyric acid (BA) and valeric acid (VA)) and BSCFA (iso‐BA, iso‐VA and isocaproic acid) of the end‐fermentation fluid were determined in these techniques.

## 2 MATERIALS AND METHODS

Ethical approval was obtained from Erciyes University Animal Experiments Local Ethics Committee (Kayseri, Türkiye) to conduct the study (Decision ID: 23/157).

### 2.1 Forage samples

As forages used in the study, alfalfa herbage (at the beginning of the flowering stage), Italian ryegrass (at the beginning of the flowering stage), meadow hay (at the full flowering stage), and wheat straw (after the seed bulking stage) were obtained from a local producer in Kayseri province (xTürkiye), approximately 5 kg each. All forages were obtained in July. After the forages were obtained, they were stored in closed containers in the laboratory to prevent moisture. None of the samples contained foreign or toxic plants. The forage samples were dried and ground in a mill with a maximum sieve diameter of 1 mm (IKA MF Basic Miller, IKA Industrie‐ und Kraftfahrzeugausrüstung GmbH) and kept in an oven at 105°C for 48 h (Nüve SN500, Nüve Laboratory and Sterilization Technology) to determine dry matter (DM) levels. The CP, diethyl ether extract, and ash contents of the samples were determined according to the Association of Official Analytical Chemists (AOAC) (1995). NDF, acid detergent fibre (ADF) and acid detergent lignin (ADL) levels were in samples, according to the methods reported in Van Soest et al. (1991). The NDF, ADF and ADL contents were determined as without ash residues (NDFom, ADFom and ADL). Chemical analyses were performed in triplicate (Table 1).

**TABLE 1:** Nutrient content of forages used in the in vitro digestion, % dry matter (DM).

	CP	EE	Ash	NSC	NDFom	ADFom	HemS	ADL	Cellulose
Alfalfa herbage	16.36	1.61	10.02	32.90	39.08	28.02	11.06	6.12	21.90
Italian ryegrass	12.43	1.70	10.67	22.03	53.15	40.88	12.27	6.81	34.07
Meadow hay	9.46	1.56	7.13	20.91	60.92	32.70	28.22	7.91	24.79
Wheat straw	4.64	1.21	14.13	10.73	69.27	45.38	23.89	11.26	34.12

*Note*: HemS (hemicellulose) = NDFom − ADFom, cellulose = ADFom − ADL.

Abbreviations: ADFom, acid detergent fibre without ash; ADL, acid detergent lignin; CP, crude protein; EE, diethyl ether extract; NDFom, neutral detergent fibre without ash; NSC, non‐structural carbohydrate.

### 2.2 In vitro digestion techniques

#### 2.2.1 Faeces inoculums

The faecal inoculums required for colonic fermentation were obtained from the same horses at the end of three different in vitro techniques. Three in vitro studies were performed synchronously. Horse faeces samples were taken from three 6‐year‐old British horses (about 650 kg of live weight) in a commercial enterprise in Talas district of Kayseri province, Türkiye. Horses had consumed a ration consisting of approximately 47.8% concentrated feeds (3.85 kg/day oats, 1.65 kg/day barley flake) + 52.2% forage (1 kg/day wheat straw, 1 kg/day English ryegrass, 2 kg/day lucerne herbage and 2 kg/day meadow grass) as DM basis. Faeces samples procured after morning feedings were taken immediately after defecation and combined under CO_2_ gas to preserve the viability of faeces’ anaerobic microorganisms in a screw‐capped glass bottle at approximately 39 ± 1°C. They were brought to the laboratory with a lidded thermos carrier. The faeces were used in the in vitro digestion technique after filtering through four layers of cheesecloth under CO_2_ gas.

### 2.3 In vitro total digestion technique

In vitro TDT in horses was performed in three stages: gastric digestion, small intestine digestion and large intestine fermentation.

- Stage (in vitro gastric digestion). The 310 ± 10 mg DM of forage was mixed with 10 mL of phosphate buffer (0.1 M, pH 6) into an anaerobic glass fermenter with a 100 mL volume (Model Fortuna, Häberle Labortechnik, Lonsee‐Ettlenschieß). In total, 5 mL 0.2 M of HCl was added to this mixture, and the pH value was adjusted to pH 2.0 (with 1 M of HCl and 1 M of NaOH). Then, 1 mL of pepsin solution (10 mg/mL) was added and incubated at 39.0 ± 0.2°C for 2 h in a thermostatic water bath (Hervera et al., 2007). At this stage, the pH value was adjusted to this value so that the pepsin enzyme could become active and initiate protein digestion.
- Stage (in vitro small intestine digestion). After the gastric digestion, 5 mL of the phosphate buffer (0.2 M, pH 6.8) and 2.5 mL of NaOH 0.6 M were added to the glass fermenters and adjusted to pH 6.8. Then, 1 mL of pancreatin solution (50 mg pancreatin/mL) was added and incubated for 4 h at 39.0 ± 0.2°C in a thermostatic water bath (Hervera et al., 2007). At this enzymatic stage, the pH value was adjusted to a value close to neutral, as in the small intestine environment, and was used to ensure that the pancreatic enzymes were active at this value and continued nutrient digestion.
- Stage (in vitro large intestine fermentation). After the in vitro small intestine digestion, the pre‐digested forages and digestion fluids were incubated with the faecal inoculum (1 mL) and fermentation medium (30 mL), which contained solution A, solution B, trace mineral solution, water‐soluble vitamins and folate: biotin solution, riboflavin solution, haemin solution, SCFAs, resazurin, yeast extract, trypticase, Na_2_CO_3_ and cysteine HCl·H_2_O (Sunvold et al., 1995), in a water bath with a thermostat set up at 39.0 ± 0.2°C for up to 24 h. In addition, six blank fermenters (no template = medium mixture plus the faecal inoculum) were used for calculations. In this large intestine simulation environment, it was aimed to ensure bacterial activation and nutrient fermentation by providing pH value with the microbial medium and end products in the large intestine.

### 2.4 In vitro Sunvold–large intestine technique

The in vitro digestion technique in horses was carried out according to Sunvold et al. (1995), which incubated feed sample in faeces inoculum and fermentation medium (Table 2). Faeces samples were diluted at a 1:10 ratio with 0.9% sterile serum physiologic solution (Polifleks, Polifarma) using a laboratory‐type blender (Waring Products Division). Diluted faeces inoculum was filtered through four layers of cheesecloth under constant CO_2_ gas (anaerobically) and used in the in vitro digestion technique. The in vitro digestion technique was conducted in glass syringes with 100 mL volume (Model Fortuna, Häberle Labortechnik, Lonsee‐Ettlenschieß) using the medium mixture, prepared according to Sunvold et al. (1995). The samples (500 ± 10 mg as DM) were incubated with the medium mixture (30 mL) and faeces inoculum (5 mL) in glass syringes (*n* = 6). The syringes were incubated in a water bath with a thermostat at 39.0 ± 0.2°C up to 24 h. In addition, six blank syringes (no template; medium mixture plus faeces inoculum) were used for calculations.

**TABLE 2:** Composition of in vitro fermentation medium.

Component	Amount
mL/L	
Solution A^a^	330.0
Solution B^b^	330.0
Trace mineral solution^c^	10.0
Water‐soluble vitamins^d^	20.0
Folate: biotin solution^e^	5.0
Riboflavin solution^f^	5.0
Haemin solution^g^	2.5
Short‐chain fatty acids^h^	0.4
Resazurin^i^	1.0
Distilled H_2_O	296.0
g/L	
Yeast extract	0.5
Trypticase	0.5
Na_2_CO_3_	4.0
Cysteine HCl·H_2_O	0.5

^a^ Composition (g/L): NaCl, 5.4; KH_2_PO4, 2.7; CaCl_2_·H_2_O, 0.16; MgCl_2_·6H_2_O, 0.12; MnCl_2_·4H_2_O, 0.06; CoCl_2_·6H_2_O, 0.06; (NH_4_)_2_SO_4_, 5.4.

^b^ Composition: K_2_HPO_4_, 2.7 g/L.

^c^ Composition (mg/L): ethylene diamine tetra acetic acid (disodium salt), 500; FeSO_4_·7H_2_O, 200; ZnSO_4_·7H_2_O, 10; MnCl_2_·4H_2_O, 3; H_3_PO_4_, 30; CoCl_2_·6H_2_O, 20; CuCl_2_·2H_2_O, 1; NiCl_2_·6H_2_O, 2; Na_2_MoO_4_·2H_2_O, 3.

^d^ Composition (mg/L): thiamin‐HCl, 100; d‐pantothenic acid, 100; niacin, 100; pyridoxine, 100; *p*‐amino benzoic acid, 5; vitamin B_12_, 0.25.

^e^ Composition (mg/L): folic acid, 10; d‐biotin, 2; NH_4_HCO_3_, 100.

^f^ Composition: riboflavin, 10 mg/L in 5 mmol/L of Hepes.

^g^ Haemin: Haemin 500 mg/L of 10 mmol/L NaOH.

^h^ Composition: *n*‐valerate, iso‐valerate, iso‐butyrate, and dl‐α‐methyl butyrate, 250 mL/L.

^i^ Composition: 1 g resazurin/L distilled water.

### 2.5 In vitro Menke–large intestine digestion technique

In vitro MDT simulates the rumen environment in ruminants and ensures digestion of feedstuffs and enzymes of microorganisms with inoculum and buffering with the chemical mixture used (Menke et al., 1979). This technique and similar techniques, such as other ruminal techniques (e.g. Karimi et al., 2022), take less time to perform and produce results with fewer chemicals; but, first, the fermentative digestion of forage in ruminants takes place, and then digestion begins in the rumen (fermentative) and continues enzymatic digestion. However, first, enzymatic digestion in horses (stomach and small intestine) and then fermentative digestion (large intestine) may indicate that it would be wrong to use this technique for two different animal species.

The forage samples were incubated in horse faeces inoculum and buffer mixture in 100 mL‐capacity calibrated anaerobic glass fermenter (Fortuna, Poulten & Graf Ltd.), following the procedures of Menke et al. (1979). The 200 ± 10 mg of dried forage samples were incubated with 20 mL of buffer mixture and 10 mL of the horse faeces inoculums mentioned above in an anaerobic glass fermenter at 39.0 ± 0.5°C in an incubator for 24 h, for six repetitions (Menke et al., 1979). One litre of buffer mixture, including 474 mL of bi‐distilled water, 237.33 mL of macro‐mineral solution (5.7 g of Na_2_HPO_4_, 6.2 g of KH_2_PO_4_ and 0.6 g of MgSO_4_ in 1 L of bi‐distilled water), 237.33 mL of buffer solution (35 g of NaHCO_3_ and 4 g of NH_4_HCO_3_ in 1 L of bi‐distilled water), 0.12 mL of trace‐mineral solution (13.2 g of CaCl_2_·2H_2_O, 10 g of MnCI_2_·4H_2_O, 1 g of CoCI_2_·6H_2_O and 0.8 g of FeCI_3_·6H_2_O in 100 mL of bi‐distilled water), 1.22 mL of resazurin solution (0.1 g of resazurin in 100 mL of bi‐distilled water) and 50 mL of reducing solution (285 mg of Na_2_S·7H_2_O and 4 mL of 1 N NaOH in 96 mL of bi‐distilled water), was added. In addition, six blank glass fermenters (no samples) were incubated to provide correction values.

### 2.6 Detection of in vitro digestion residues

In all three techniques, at the end of incubation, the undigested feed residue was taken from the syringe and passed through 95% ethanol, absolute acetone and 75% ethanol, respectively, to remove lipids and free sugars. This process was performed using a vacuum unit (Velp Dietary Fiber Analyzer, Velp Scientifica) on pre‐weighed glass crucibles (Velp, porosity 2). After these washing steps, the crucibles were dried overnight at 105°C, weighed and recorded, and in vitro T‐DMD was determined. Then, three glass crucibles were burned in a muffle furnace at 550°C for a minimum of 8 h, and in vitro T‐OMD was calculated. The NDFom level for each forage sample in each in vitro technique in the other three glass crucibles was determined by Van Soest et al. (1991) and detected in vitro T‐NDFD:

(1) $$\begin{array}{ccc} & & \mathrm{In}\mathrm{vitro}T-\mathrm{DMD}\mathrm{was}\mathrm{calculated}\mathrm{as}1\\ & & \;-\left[\left(\mathrm{DM}\mathrm{residue}-\mathrm{DM}\mathrm{blank}\right)/\mathrm{initial}\mathrm{DM})\right]\times 100 \end{array}$$

(2) $$\begin{array}{ccc} & & \mathrm{In}\mathrm{vitro}T-\mathrm{OMD}\mathrm{was}\mathrm{calculated}\mathrm{as}1\\ & & \;-\left[\left(\mathrm{OM}\mathrm{residue}-\mathrm{OM}\mathrm{blank}\right)/\mathrm{initial}\mathrm{OM})\right]\times 100 \end{array}$$

(3) $$\begin{array}{ccc} & & \mathrm{In}\mathrm{vitro}T-\mathrm{NDFD}\mathrm{was}\mathrm{calculated}\mathrm{as}1\\ & & \;-\left[\left(\mathrm{NDFom}\mathrm{residue}-\mathrm{NDFom}\mathrm{blank}\right)/\mathrm{initial}\mathrm{NDFom})\right]\times 100. \end{array}$$

### 2.7 Determination of short‐chain fatty acid composition and pH in the in vitro digestion fluid

SCFAs, the end products of fermentation of fibrous compounds/dietary fibre substances (cellulose, hemicellulose, pectin, beta‐glucan, resistance starch etc.) by the anaerobic microbiota of large intestine, have been shown to exert multiple beneficial effects on mammalian energy metabolism (Den Besten et al., 2013; Kara et al., 2022; Kara, 2022). The BSCFAs, for example iso‐BA and iso‐VA, are generated by the fermentation of branched amino acids, generated from undigested protein reaching colon and also have effects on adipocyte lipid and glucose metabolism (Heimann et al., 2016). The pH levels of the incubation fluids (15 mL) obtained at the end of in vitro digestion techniques were determined with a digital pH meter (Mettler Toledo S220 pH/ion meter, Mettler‐Toledo Ltd.). Acid compositions of in vitro digestion fluid (SCFA) [AA (C2:0), PA (C3:0), BA (C4:0),VA (C5:0), hexanoic acid (C6:0), heptanoic acid (C7:0)] (BSCFA) [iso‐butyric acid – IBA (C4:0i), iso‐valeric acid – IVA (C5:0i) and isocaproic acid – ICA (C6:0i)] were detected in gas chromatography device (GC‐FID) (TRACE1300, Thermo Fisher Scientific) with flame ion detector‐FID. A polyethylene glycol column (length: 60 m, i.d: 0.25 mm, film thickness: 0.25 μm; TG‐WAXMS, Thermo Scientific) was used for this analysis in the device, and the analysis procedure was applied according to Ersahince and Kara (2017). The compositions of total SCFA (T‐SCFA = SCFA + BSCFA) and individual SCFAs (mmol/L and %) and the AA/PA ratio (A/P) and (AA + BA)/(PA) ratio ((A + B)/P) were determined.

### 2.8 Statistical analysis

The statistical analyses of the data were performed using the SPSS 17.0 software (IBM Corp.). The experimental data were first subjected to Levene's test to detect the variance homogeneity.

All the in vitro digestion data were checked for normality using the Shapiro–Wilk test. Normal distribution of data considered for *p* > 0.05.

The effect of different forages and different in vitro digestion techniques of the analysed variables was determined with a two‐way analysis of variance in the SPSS 17.0 package program (IBM Corp.). When statistical significance was determined, Tukey's multiple comparison test was applied. The significance level was taken as *p* < 0.05.

## 3 RESULTS

### 3.1 In vitro T‐DMD, T‐OMD and T‐NDFD values

**FIGURE 1:**
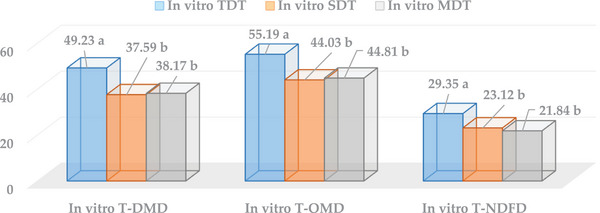
The average in vitro true dry matter digestion (T‐DMD), true organic matter digestion (T‐OMD) and true neutral detergent fibre digestion (T‐NDFD) values (%) of forages as the average of different digestion techniques. Statistical significance among in vitro T‐DMD, T‐OMD and T‐NDFD values (%) for the technique is indicated by lower lettering (^a,b^).

Differences in the digestion residues were determined between the in vitro digestion techniques. For the four forages, the in vitro TDT had the highest in vitro T‐DMD, T‐OMD and T‐NDFD values (*p* < 0.05) (Figure 1). In the present study, in vitro T‐DMD (65.88%) and T‐OMD (70.48%) values of alfalfa herbage were found to be higher than those of Italian ryegrass (51.07% and 58.15%), meadow hay (45.77% and 49.80%) and wheat straw (34.20% and 42.31%) in the in vitro TDT (*p* < 0.001). At the same time, the in vitro T‐DMD and T‐OMD values of Italian ryegrass and meadow hay were higher than that of wheat straw in the in vitro TDT (*p* < 0.001). The in vitro T‐NDFD values of alfalfa herbage for the in vitro TDT, SDT, and MDT were higher than those of other forage (*p* < 0.05). In vitro TDT and T‐NDFD of Italian ryegrass and meadow hay were higher than that of wheat straw (*p* < 0.05). In general, forages were ranked as alfalfa hay > Italian grass > meadow hay = wheat straw according to the in vitro T‐NDFD values of the three methods (*p* < 0.05). The highest in vitro T‐NDFD ratio of the forages studied in these three methods was found in the in vitro TDT (29.35%) (*p* < 0.05) (Table 3).

**TABLE 3:** In vitro true dry matter digestion (T‐DMD), true organic matter digestion (T‐OMD) and true neutral detergent fibre digestion (T‐NDFD) values of different forages in horses for in vitro digestion techniques (%).

Techniques	Forages	In vitro T‐DMD	In vitro T‐OMD	In vitro T‐NDFD
In vitro TDT	Alfalfa herbage	65.88^A^	70.48^A^	42.39^A^
	Meadow hay	45.77^B^	49.80^B^	25.19^B^
	Italian ryegrass	51.07^B^	58.15^B^	28.84^B^
	Wheat straw	34.20^C^	42.31^C^	20.97^C^
In vitro SDT	Alfalfa herbage	50.51^A^	55.43^A^	34.88^A^
	Meadow hay	33.80^B^	38.55^BC^	14.83^C^
	Italian ryegrass	37.86^B^	47.42^AB^	28.72^B^
	Wheat straw	28.19^C^	34.73^C^	14.03^C^
In vitro MDT	Alfalfa herbage	48.35^A^	53.57^A^	29.67^A^
	Meadow hay	35.17^B^	38.81^B^	17.66^C^
	Italian ryegrass	45.74^A^	55.82^A^	23.68^B^
	Wheat straw	23.41^B^	31.05^B^	16.35^C^
Forage	Alfalfa herbage	54.91^x^	59.82^x^	35.65^x^
	Meadow hay	38.25^z^	42.39^z^	20.23^z^
	Italian ryegrass	44.89^y^	53.80^y^	27.08^y^
	Wheat straw	28.59^q^	36.02^q^	17.12^z^
Technique	In vitro TDT	49.23^a^	55.19^a^	29.35^a^
	In vitro SDT	37.59^b^	44.03^b^	23.12^b^
	In vitro MDT	38.17^b^	44.81^b^	21.84^b^
SD		11.95	11.59	8.69
*p* Values	Technique	<0.001	<0.001	<0.001
	Forage	<0.001	<0.001	<0.001
	Technique × forage interactions	0.114	0.085	0.016

*Note*: Statistical significance for the technique is indicated by lower lettering (^a,b^). Statistical significance for forage types is indicated by lower lettering (^x,y,z,q^). Upper letters (^A,B,C^) indicate that the statistical significance of forage types according to different methods and two‐way ANOVA is shown with these assumptions.

Abbreviations: in vitro MDT, Menke–large intestine digestion technique; in vitro SDT, in vitro Sunvold–large intestine technique; in vitro T‐DMD, in vitro true dry matter digestion, %; in vitro TDT, in vitro total digestion technique; in vitro T‐NDFD, in vitro true neutral detergent fiber digestion; in vitro T‐OMD, in vitro true organic matter digestion, %; SD, standard deviation of means.

According to the in vitro SDT, the in vitro T‐DMD (50.51%) and T‐OMD (55.43%) values of alfalfa herbage were found to be higher than those of meadow hay (33.85% and 38.55%) and wheat straw (28.19% and 34.73%) (*p* < 0.001). The in vitro T‐DMD and T‐OMD of meadow hay and Italian grass were similar according to the in vitro SDT (*p* > 0.05) (Table 3).

**FIGURE 2:**
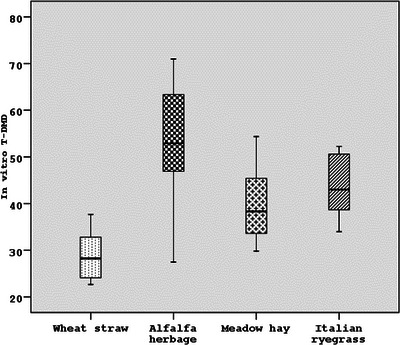
The in vitro true dry matter digestion (T‐DMD) values (%) of forages as the average of four different digestive techniques.

**FIGURE 3:**
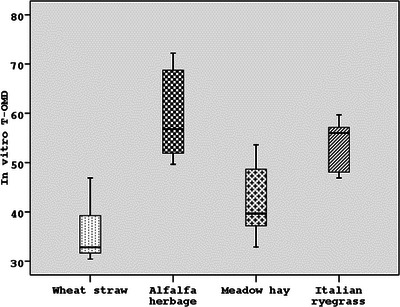
The in vitro true organic matter digestion (T‐OMD) values (%) of forages as the average of four different digestive techniques.

**FIGURE 4:**
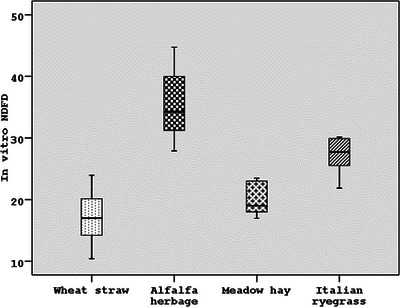
The in vitro true neutral detergent fibre digestion (T‐NDFD) values (%) of forages as the average of four different digestive techniques.

In the in vitro MDT in horses, alfalfa herbage (48.35%) and Italian ryegrass (45.74%) had a higher in vitro T‐DMD value according to those of meadow hay (35.17%) and wheat straw (23.41%) (*p* < 0.001). According to the same method, the in vitro T‐OMD values of alfalfa herbage (53.57%) and Italian grass (55.82%) were higher than those of wheat straw (31.05%) and meadow hay (38.81%) (*p* < 0.001) (Table 3). When the in vitro digestion levels of forage were compared, the in vitro T‐DMD and T‐OMD levels of alfalfa herbage (54.91% and 59.82%, respectively) and Italian grass (44.89% and 53.80%, respectively) were found to be higher than those of meadow hay (38.25% and 53.80%, respectively) and wheat straw (28.59% and 36.02%, respectively) (*p* < 0.001) (Table 3) (Figures 2, 3, 4).

Generally, when the effects of the in vitro digestion techniques on the digestion levels of forages were compared, in vitro T‐DMD and T‐OMD levels of forage were higher in the in vitro TDT than the in vitro SDT and in vitro MDT (*p* < 0.001) (Table 3).

### 3.2 Digestion fluid short‐chain fatty acids concentrations

In the in vitro TDT, the molarities of AA, T‐SCFA and BSCFA in the digestion fluid of alfalfa herbage were higher than those of other forages (*p* < 0.05). In this technique, the forage was ranked as alfalfa herbage > meadow hay = Italian ryegrass > wheat straw according to the AA molarity (*p* < 0.05). In the in vitro TDT, the VA molarities in alfalfa herbage and Italian ryegrass were higher than in the other forages (*p* < 0.05). According to the three methods, at the end of the in vitro digestion of the forages, the highest molarities of AA, PA, BA, and VA were found in the in vitro TDT (*p* < 0.05). The molarities of AA and BA of alfalfa herbage were higher than those of other forages (*p* < 0.05) (Tables 4 and 5).

**TABLE 4:** Short‐chain fatty acids concentrations in the in vitro digestion fluid of forage by different in vitro digestion techniques in horses.

Techniques	Forages	BSCFA			SCFA			
		ICA	IBA	IVA	VA	BA	PA	AA
In vitro TDT	Alfalfa herbage	0.23^A^	0.27^A^	0.25^A^	0.26^A^	8.70	26.19	41.84^A^
	Meadow hay	0.21^B^	0.25^B^	0.23^B^	0.24^B^	8.62	26.07	40.55^B^
	Italian ryegrass	0.23^A^	0.28^A^	0.27^A^	0.25^A^	8.61	26.11	40.97^B^
	Wheat straw	0.21^B^	0.24^B^	0.22^B^	0.20^C^	8.04	25.53	39.87^C^
In vitro SDT	Alfalfa herbage	0.21	0.30	0.30	0.23	8.26	24.90	42.00
	Meadow hay	0.21	0.29	0.27	0.23	7.99	24.88	40.61
	Italian ryegrass	0.21	0.29	0.30	0.23	7.78	24.86	39.51
	Wheat straw	0.21	0.29	0.25	0.21	7.72	24.82	39.30
In vitro MDT	Alfalfa herbage	0.21	0.22	0.19	0.19^B^	7.72	24.74	38.74
	Meadow hay	0.21	0.23	0.19	0.19^B^	7.71	24.74	38.74
	Italian ryegrass	0.21	0.24	0.21	0.22^A^	7.73	24.75	38.76
	Wheat straw	0.21	0.25	0.20	0.19^B^	7.71	24.74	38.80
Forage	Alfalfa herbage	0.21	0.26	0.25	0.23^x^	8.23^x^	25.27	40.86^x^
	Meadow hay	0.21	0.25	0.23	0.22^xy^	8.11^y^	25.23	39.96^y^
	Italian ryegrass	0.21	0.27	0.26	0.23^x^	8.04^y^	25.24	39.75^y^
	Wheat straw	0.21	0.26	0.22	0.20^y^	7.82^z^	25.03	39.32^y^
Technique	In vitro TDT	0.21	0.26^b^	0.24^b^	0.24^a^	8.49^a^	25.97^a^	40.81^a^
	In vitro SDT	0.21	0.29^a^	0.28^a^	0.23^b^	7.94^b^	24.86^b^	39.15^b^
	In vitro MDT	0.21	0.24^c^	0.20^c^	0.20^c^	7.72^c^	24.74^c^	38.76^b^
SD		0.001	0.03	0.04	0.02	0.41	0.59	1.33
*p* Values	Technique	0.251	<0.001	<0.001	<0.001	<0.001	<0.001	<0.001
	Forage	0.061	0.045	0.024	<0.001	<0.001	<0.001	0.002
	Technique × forage interactions	0.051	0.055	0.280	<0.001	0.012	<0.001	0.055

*Note*: This lower letter (^a,b,c^) indicates statistical significance for the technique. Statistical significance for forage types is indicated by lower lettering (^x,y,z^). Upper latter (^A,B,C^) indicates that the statistical significance of forage types according to different methods and two‐way ANOVA is shown with these assumptions.

Abbreviations: AA, acetic acid; BA, butyric acid; BSCFA, branched short‐chain fatty acid (ICA + IBA + IVA); IBA, iso‐butyric acid; ICA, isocaproic acid; in vitro MDT, Menke–large intestine digestion technique; in vitro SDT, In vitro Sunvold–large intestine technique; In vitro TDT, in vitro total digestion technique; IVA, iso‐valeric acid; PA, propionic acid; SCFA, straight short‐chain fatty acid (AA + BA + PA + VA); SD, standard deviation of means; VA, valeric acid.

**TABLE 5:** Calculated short‐chain fatty acid concentrations and pH values in the in vitro digestion fluid of forage by different in vitro digestion techniques in horses.

Techniques	Forages	A/P	(A + B)/P	T‐SCFA	BSCFA	SCFA	pH
In vitro TDT	Alfalfa herbage	1.60^A^	1.93^A^	77.71^A^	0.73^B^	76.98^A^	7.71
	Meadow hay	1.56^B^	1.89^B^	76.16^B^	0.69^C^	75.48^B^	7.48
	Italian ryegrass	1.57^A^	1.90^A^	76.70^B^	0.76^A^	75.94^B^	7.92
	Wheat straw	1.56^B^	1.88^B^	74.31^C^	0.67^C^	73.64^C^	8.35
In vitro SDT	Alfalfa herbage	1.69	2.02	76.20	0.81	75.39	6.93
	Meadow hay	1.63	1.95	74.47	0.77	73.70	7.48
	Italian ryegrass	1.59	1.90	73.18	0.79	72.39	7.66
	Wheat straw	1.58	1.89	72.80	0.75	72.05	8.53
In vitro MDT	Alfalfa herbage	1.57	1.88	72.01	0.62	71.39	6.65
	Meadow hay	1.57	1.88	71.99	0.62	71.38	6.67
	Italian ryegrass	1.57	1.88	72.12	0.66	71.47	6.68
	Wheat straw	1.57	1.88	72.10	0.66	71.44	7.32
Forage	Alfalfa herbage	1.62	1.94	75.31	0.72	74.59	7.10^y^
	Meadow hay	1.58	1.91	74.21	0.69	73.52	7.21^y^
	Italian ryegrass	1.57	1.89	74.00	0.74	73.26	7.42^y^
	Wheat straw	1.57	1.88	73.07	0.69	72.38	8.06^x^
Technique	In vitro TDT	1.57^b^	1.90^b^	76.22^a^	0.71^b^	75.51^a^	7.86^a^
	In vitro SDT	1.62^a^	1.94^a^	74.16^b^	0.78^a^	73.38^b^	7.65^a^
	In vitro MDT	1.57^b^	1.88^b^	72.06^c^	0.64^c^	71.42^c^	6.83^b^
SD			0.05	2.18	0.07	2.15	0.73
*p* Values	Technique	<0.001	<0.001	<0.001	<0.001	<0.001	<0.001
	Forage	0.011	0.009	<0.001	0.024	<0.001	0.001
	Technique × forage interactions	0.057	0.075	0.028	0.173	0.032	0.590

*Note*: This upper letters (^a,b,c^) indicate statistical significance for the technique. Statistical significance for forage types is indicated by lower lettering (^x,y^). Upper letters (^A,B,C^) indicate that the statistical significance of forage types according to different methods and two‐way ANOVA is shown with these assumptions.

Abbreviations: BSCFA, branched short‐chain fatty acid; in vitro MDT, Menke–large intestine digestion technique; in vitro SDT, in vitro Sunvold–large intestine technique; in vitro TDT, in vitro total digestion technique; SCFA, straight short‐chain fatty acid; SD, standard deviation of means; T‐SCFA, total straight short‐chain fatty acid.

In the in vitro TDT, the ICA, IBA, IVA, A/P and (A + B)/P concentrations in the digestion fluid differed, and higher values were found for alfalfa herbage and Italian ryegrass (*p* < 0.05). The BSCFA molarities did not differ between forages in the in vitro SDT and MDT (*p* < 0.05). There was a difference between the methods in terms of iso‐acid molarities, and the highest BSCFA values were found in the in vitro SDT according to the in vitro SDT and MDT (*p* < 0.05) (Tables 4 and 5).

### 3.3 Pearson (r) correlations of forage digestion variables with different in vitro digestion methods in horses

The study determined a negative correlation between the NDFom, ADFom and cellulose levels of the forage and the in vitro T‐DMD, T‐OMD and T‐NDFD levels in horses (*p* < 0.05). In the present study, there was no correlation between the ICA and IBA levels of the in vitro fermentation fluid of forages and the in vitro T‐DMD and T‐NDFD (*p* > 0.05). There was a positive correlation among the in vitro T‐DMD, T‐OMD and T‐NDFD levels of forages and AA and BA concentrations of the in vitro fermentation fluid (*p* < 0.05) (Table 6).

**TABLE 6:** Pearson (*r*) correlations of forage’ digestion variables for different in vitro digestion techniques in horses.

	In vitro T‐DMD	In vitro T‐OMD	In vitro T‐NDFD
In vitro T‐OMD	0.982^**^	1	0.860^**^
In vitro T‐NDFD	0.860^**^	0.858^**^	1
NDFom	−0.761^**^	−0.793^**^	−0.838^**^
ADFom	−0.632^**^	−0.516^**^	−0.554^**^
Cellulose	−0.522^**^	−0.369^*^	−0.433^**^
ICA	−0.003	−0.093	−0.047
VA	−0.113	−0.201	−0.279
IBA	−0.047	−0.022	−0.053
IVA	−0.153	−0.224	−0.133
BA	0.313^*^	0.308^*^	0.349^*^
PA	−0.108	−0.074	−0.157
AA	0.393^**^	0.358^**^	0.402^**^

Abbreviations: AA, acetic acid; ADFom, acid detergent fibre without ash; BA, butyric acid; IBA, iso‐butyric acid; ICA, isocaproic acid; in vitro T‐DMD, in vitro true dry matter digestion; in vitro T‐NDFD, in vitro true neutral detergent fiber digestion; in vitro T‐OMD, in vitro true organic matter digestion; IVA, iso‐valeric acid; NDFom, neutral detergent fibre without ash, PA, propionic acid; VA, valeric acid.

** Correlation is significant at the 0.01 level.

* Correlation is significant at the 0.05 level.

## 4 DISCUSSION

In equids, fibrous compounds can be fermented by microbial enzyme activity in the colon (Frape, 2004; NRC, 2007). However, in addition to the fibrous carbohydrates in the forage, core nutrients such as CP and NSC are theoretically digested by enzymes such as proteolytic and amylolytic enzymes in the stomach and small intestine after the horse consumes the feed. Afterwards, the feed material, whose enzymatic digestion has been completed, only reaches the large intestine and is fermented by microbial fibrolytic enzymes (Dicks et al., 2014). In vitro techniques simulated for in vitro forage digestion in horses were based on colonic fibre fermentation (Menke et al., 1979; Theodorou et al., 1994). It seems that starting directly with colon fermentation for fibre digestion in horses may be wrong, according to the results of the present study. In the present study, the highest in vitro digestion levels (T‐DMD, T‐OMD and T‐NDFD) for the four different forages examined were detected in the in vitro TDT, a digestion simulation of the entire gastrointestinal tract. The high digestion level of forages in the in vitro TDT showed that its effectiveness in forage digestion and fermentation would yield better results than techniques that only simulate colon fermentation techniques (in vitro SDT and MDT). The difference between the in vitro digestion results of the techniques also created differences in the forage types. The alfalfa herbage reached the highest values in horses with approximately 71% of in vitro T‐OMD, 66% of in vitro T‐DMD and 42% of in vitro T‐NDFD levels in horses with the in vitro TDT, which was related to its lower ADF level and higher NSC value than the other researched forages (Ersahince & Kara, 2017; Kara, Güçlü, et al., 2022; Kara, 2022; Kara, Yılmaz, et al., 2022).

In a horse's diet, sugars from NSC substances are absorbed directly from the small intestine and non‐resistance starch, and diglycerides from NSC substances are digested by enzymes in the small intestine and absorbed as monomers (Frape 2004; NRC, 2007). If these substances are not digested well or the horse consumes too many NSC substances in their diet, higher amounts of these substances may reach the colon and cause increased acidity and then ulcers (Colombino et al., 2022; Raspa, Tarantola et al., 2022; Raspa, Vervuert, et al., 2022). The acidity value (pH 6.83) of forage in the in vitro MDT was higher according to that of the in vitro TDT (pH 7.86) which may be due to the NSC elements that did not undergo in vitro intestinal digestion and were present in the fermentation environment. The prececal digestion of mono and diglycerides (especially fructan structures), which is an important ingredient of the NSC element in forage, does not reach the colon fermentation too much (Moore‐Colyer et al., 2013; Strauch et al., 2017), showing that forage with a high NSC value (especially alfalfa herbage) could be digested better with the in vitro TDT. Likewise, the in vitro T‐DMD, T‐OMD and T‐NDFD levels of Italian ryegrass and meadow hay were higher than those of wheat straw in the in vitro TDT, which may be associated with the high NDF and high lignocellulose complex in the straw (NRC, 2001), a forage obtained at the end of the plant's phenological period. The in vitro digestion levels of the analysed forage differed in methods and among themselves. In general, ranking the forages as alfalfa herbage > Italian ryegrass > meadow hay = wheat straw, according to the in vitro T‐NDFD levels in the three methods, was thought to be related to the water‐soluble carbohydrates (WSC) and lignocellulose complex contents of these forages (Kara & Baytok, 2017; Murray et al., 2006; Stang et al., 2022). The starch content in the hay or straw of legumes and grasses, which is widely used in horses, is between 1% and 3% in DM (NRC, 2007). Our study found that the highest NSC and WSC contents are in alfalfa herbage and Italian ryegrass, according to those of other researched forages. Similar to the present study results, Stang et al. (2022) reported that the in vitro T‐DMD value for six grass species (meadow fescue, cocksfoot, perennial ryegrass, smooth bromegrass, tall fescue and timothy for three harvest times: early, medium and late first cut) was between 60% and 64%. The same researcher reported that the in vitro T‐DMD value reduced from about 70% to 54% with a decrease in the level of WSC (glucose and fructose), an increase in the NDF and a decrease in the WSC (glucose and fructose) levels in forage during harvest time. The alfalfa herbage had higher in vitro T‐DMD and T‐OMD levels than other forages in the in vitro TDT, which can also be associated with the enzymatic digestion of CP and NSC, which are the total digestible nutrients, in the stomach and small intestine (Hervera et al., 2007). Although there is very little information about the taste of feed on feed preference in horses, factors that will increase the taste of forage can change the preference of horses (Vinassa et al., 2020). In the present study, alfalfa herbage and Italian ryegrass are superior in terms of digestion capacity in in vitro digestion in horses, and their high content of soluble carbohydrates may have properties that may increase horse preference.

Previous studies have not compared in vitro digestion techniques in horses. However, the results obtained in some in vitro techniques may help compare the results of the techniques in the present study. Cagri and Kara (2018) reported that horse rations containing safflower grain, safflower straw and safflower hay using the in vitro SDT in horses had approximately 44%–50% in vitro T‐DMD and 45–56 of T‐OMD levels. Similarly, adding up to 20% tomato pulp as a soluble fibre source in horse rations increased the in vitro T‐DMD and the SCFA concentration with the in vitro SDT (Kara, 2022). In a technique similar to the in vitro MDT (Theodorou et al., 1994), they reported that DM loss (from 466 to 658 mg/g) increased with the increase in sugar beet in the feed mixture in horses (Murray et al., 2006). In another technique similar to the in vitro MDT with the ANKOM RF gas production system (Goering & Van Soest, 1970). Stang et al. (2022) stated that the in vitro T‐DMD value for six grass species was between 60% and 64%. Therefore, Ersahince and Kara (2017), using the in vitro SDT, reported that the in vitro T‐DMD of Jerusalem artichoke in different phenological periods had approximately 43%–58% of in vitro T‐DMD and was 48%–65% of the in vitro T‐OMD.

In the case of relatively low starch and high structural carbohydrates in the cecum and colon, the SCFA that develops there is mainly AA. The fibrolytic bacteria colonizing here are *Bacteroides*, *Bifidobacterium, Eubacterium*, *Propionibacterium*, *Selenomonas* and *Streptococcus* (Costa et al., 2012; Mackie & Wilkins, 1988). The increase in the concentration of SCFAs obtained by the fermentation of forage in horses indicates high digestibility (Ersahince & Kara, 2017). The molarities of AA, PA and BA for three different digestion techniques applied in horses in the current study were similar to the results of previous studies (Ersahince & Kara, 2017). The positive correlation between the levels of the in vitro T‐DMD, T‐OMD and T‐NDFD of forage and the concentrations of AA and PA in the digestion fluid in the present study was consistent with the results of previous studies (Ersahince & Kara, 2017; Kara et al., 2022). However, the lack of a correlation between the concentration of BSCFA in the digestion fluid and the levels of the in vitro T‐DMD and T‐NDFD of forage in the present study was thought to be related to the fact that iso‐acids are primarily associated with protein breakdown, and the CP value of forage is not very high. They mainly consist of the iso‐acids degradation products of valine, isoleucine, leucine and proline amino acids and require their use for the biosynthesis of these amino acids and higher BSCFA, respectively (Apajalahti et al., 2019). In addition, in the present study, a difference was found in the BSCFA molarity of forage in the in vitro TDT, and the highest ICA, IBA and IVA concentrations were found in alfalfa herbage and Italian ryegrass with the highest CP content.

Some methods, such as those in ruminants (Kara et al., 2022; Menke et al., 1979; Theodorou et al., 1994), have been used to predict the in vitro digestibility of feed consumed by horses. However, these methods are generally modified versions developed on ruminants. The microbial profile and fermentation events in the colon of horses and the end products of fermentation are similar to the fermentative activity in the rumen of ruminates (Cagri & Kara, 2018; Costa et al., 2012; Ersahince & Kara, 2017; Mackie & Wilkins, 1988). However, most of the nutrients (such as protein, fat, mono‐ and di‐saccharides and non‐resistant starch) in the feedstuffs are broken down and absorbed by the organism's enzymes in the stomach and small intestine by enzymatic (hydrolysis) digestion until they reach the colon in horses. The indigestible structural carbohydrates which reach the colon and the feedstuffs after being pre‐digested/processed for fermentation and wait for fermentation are significantly different for forage fermentation. In the present study, it was understood that the methods using the rumen content (Menke et al., 1979; Theodorou et al., 1994), which were converted to a modified horse digestion method with inoculum including horse faeces (Gandarillas et al., 2021; Kujawa et al., 2020; Kholif et al., 2015; Stang et al., 2022), may be inaccurate for forage digestion. According to the present study results, it is wrong to think that the digestion of forage materials is completed only in colon fermentation. A technique including the in vitro stimulation of enzymatic digestion and microbial fermentation in the total gastrointestinal tract has shown better results. It can be predicted that different forages will have different in vitro digestion values. However, in the present study, the differences between forages with different CP, NSC and NDF contents were revealed with enzymatic + fermentative digestion or only fermentative digestion. In this study, it was revealed that the in vitro OMD, in vitro DMD and in vitro NDFD values obtained by the in vitro TDT for alfalfa herbage, which had the highest CP and NSC values among the forages studied, were 40%–50% higher than the in vitro SDT and in vitro MDT values. However, the lack of a high level of difference in wheat straw compared to alfalfa herbage was thought to be due to the fact that the content of this forage largely consists of NDF. In a study investigating in vitro total digestion in horses (Gandarillas et al., 2021), it was demonstrated that gas production and SCFA of concentrates and grains were higher than those of forages when evaluated alone. Previous in vitro digestion, which was based on only colonic fermentation using donkey faeces as a source of microbial inoculum, reported that alfalfa herbage and Italian ryegrass reached approximately 30% in vitro DMD, and wheat straw reached approximately 10% in vitro DMD at the 30th hour of incubation (Tassone et al., 2019). The same researchers (Tassone et al., 2019), using an in vitro technique based on colonic fermentation, reported that the in vitro NDFD of wheat straw, Italian ryegrass and alfalfa herbage were <5%, about 10% and about 15%, respectively. As can be seen from the studies, it is obvious that the in vitro total digestion for forage and concentrates will give more accurate results. A system called the GastroIntestinal Model had also been developed to understand the total digestion of young, adult and elderly humans, and dogs, pigs and calves after the ingestion of various meals (Minekus, 2015). However, the cost of this system shows that it would be more logical to perform each stage of this process step by step in the present study.

## 5 CONCLUSIONS

Starting directly with simulated colon fermentation for in vitro forage digestion treatment in horses may be wrong. The desired in vitro forage digestion is provided, first, with the digestion of CP, NSC and other elements by the stomach and small intestine enzymes, just as it occurs in the in vivo environment, and there should be no NSC elements (or at a low rate) that will lower the pH in the fermentation environment. The horse's in vitro total enzymatic + fermentative digestion (in vitro TDT) technique revealed higher values than the in vitro fermentative digestion techniques (in vitro MDT and in vitro SDT). Generally, the forage with higher NSC and CP levels had higher digestion values for the in vitro TDT than the other techniques. In addition, as the NDFom, ADFom and cellulose contents of the forages increased, the in vitro T‐DMD, T‐OMD and T‐NDFD levels of the forage commonly used in horses decreased. In addition to the fact that all these results are remarkable and guide the researchers, there is a need for more extensive studies to compare these in vitro results with those obtained in future in vivo studies of horses.

## AUTHOR CONTRIBUTIONS

*Conceptualization; data curation; formal analysis; funding acquisition; investigation; methodology; project administration; resources; software; supervision; validation; visualization; writing – original draft; writing – review and editing*: Kanber Kara. *Data curation; formal analysis; investigation*: Abdullah Altınsoy.

## CONFLICT OF INTEREST STATEMENT

The authors declare no conflicts of interest.

## FUNDING INFORMATION

This study was supported by the Research Fund of Erciyes University (Kayseri, Türkiye) with Project ID: TYL‐2022‐11824. This study is summarized from Abdullah Altinsoy's master's thesis with the same name in title.

## ETHICS STATEMENT

The authors confirm that the ethical policies of the journal, as noted on the journal's author guidelines page, have been adhered to, and the appropriate ethical review committee approval was received. The authors confirm that they followed Erciyes University standards to protect animals for scientific purposes and feed legislation.

## CONSENT FOR PUBLICATION

All authors have consented to the publication and presentation of the data in this article.

## INSTITUTIONAL REVIEW BOARD STATEMENT

The authors confirm that the ethical policies of the journal, as noted on the journal's author guidelines page, have been adhered to, and the appropriate ethical review committee approval was received. The study was conducted in accordance with the Declaration of Helsinki, and approved by the Erciyes University Animal Experiment Ethics Committee (Decision ID: 23/157).

## Data Availability

The data analysed in this investigation are available upon request to the corresponding author.
